# Estimating the location and size of retinal injections from orthogonal images of an intact retina

**DOI:** 10.1186/s12868-015-0217-8

**Published:** 2015-11-21

**Authors:** J. J. Johannes Hjorth, Elise Savier, David C. Sterratt, Michaël Reber, Stephen J. Eglen

**Affiliations:** Cambridge Computational Biology Institute, University of Cambridge, Wilberforce Road, Cambridge, CB3 0WA UK; Institute of Cellular and Integrative Neuroscience, CNRS UPR 3212, 5 rue Blaise Pascal, 67084 Strasbourg, France; Institute for Adaptive and Neural Computation, School of Informatics, University of Edinburgh, 10 Crichton Street, Edinburgh, EH8 9AB UK; Universitéde Strasbourg Institut d’Études Avancées, 5 rue Blaise Pascal, 67084 Strasbourg, France

**Keywords:** Retinotopic mapping, Retinal injection, Native coordinate system

## Abstract

**Background:**

To study the mapping from the retina to the brain, typically a small region of the retina is injected with a dye, which then propagates to the retina’s target structures. To determine the location of the injection, usually the retina is dissected out of the eye, flattened and then imaged, causing tears and stretching of the retina. The location of the injection is then estimated from the image of the flattened retina. Here we propose a new method that avoids dissection of the retina.

**Results:**

We have developed IntactEye, a software package that uses two orthogonal images of the intact retina to locate focal injections of a dye. The two images are taken while the retina is still inside the eye. This bypasses the dissection step, avoiding unnecessary damage to the retina, and speeds up data acquisition. By using the native spherical coordinates of the eye, we avoid distortions caused by interpreting a curved structure in a flat coordinate system. Our method compares well to the projection method and to the Retistruct package, which both use the flattened retina as a starting point. We have tested the method also on synthetic data, where the injection location is known. Our method has been designed for analysing mouse retinas, where there are no visible landmarks for discerning retinal orientation, but can also be applied to retinas from other species.

**Conclusions:**

IntactEye allows the user to precisely specify the location and size of a retinal injection from two orthogonal images taken of the eye. We are solving the abstract problem of locating a point on a spherical object from two orthogonal images, which might have applications outside the field of neuroscience.

## Background

The connections from the retina are topographically organised into maps in the brain, meaning nearby cells in the retina project to neighbouring cells in each target structure [1]. To study the connectivity of the retina and its targets we need a reliable system to specify retinal locations precisely. This is complicated by a lack of visible retinal landmarks that are consistent between individuals. Furthermore, the curvature of the eye means we cannot simply represent a location on the retina in Cartesian coordinates, as this would lead to distortions.

**Fig. 1:**
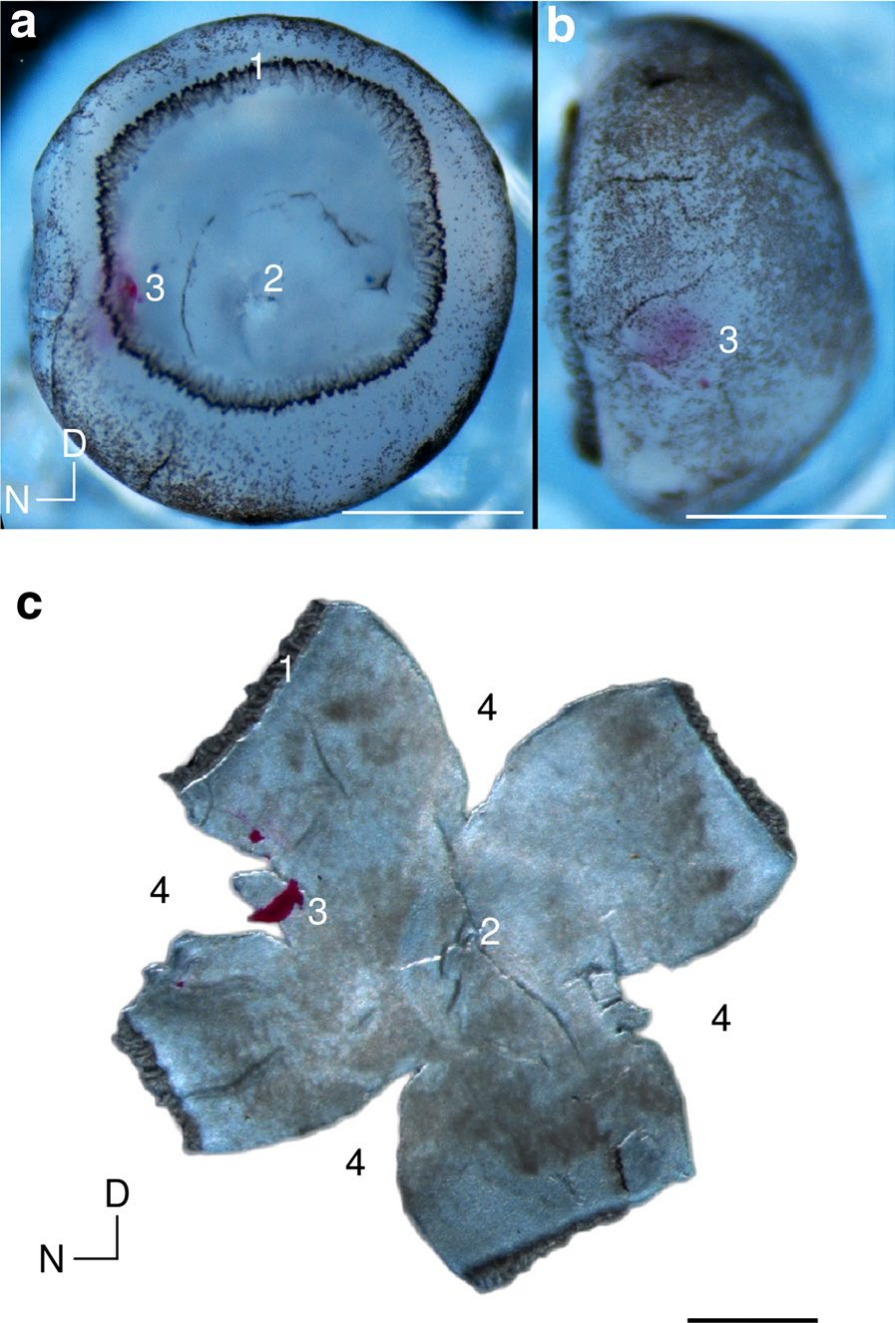
Example mouse eye at postnatal day P8 with a retinal injection. **a**, **b** Bright-field images of the intact retina, view from top and the side. *1* Retinal rim. *2* optic disc. *3* dye injection. **c** Flattened retina. Four nasal, temporal, dorsal and ventral cuts (*4*) were made to flatten the retina. The injection site is visible as a *claret-coloured mark* (*3*) on the retina. *Scale bar* 1 mm. Nasal (N) and Dorsal (D) directions are marked

The retinal projections to the brain have been studied extensively as a model system for self-organisation of the brain [1–3]. The most widely-used technique to analyse retinotopy is to inject a dye into a small region of the eye to label a region of the retina (Fig. 1A, B). The dye is then transported through retinal axons to label target structures [4–6]. Although this method is over twenty five years old [7], it is still commonly used today [8]. A common alternative for assessing retinotopic map order is to use imaging techniques [9]. However, these imaging methods require the retina to generate visual responses, around postnatal day 10 in mouse, by which time the mouse retinocollicular map has already been established.

**Fig. 2:**
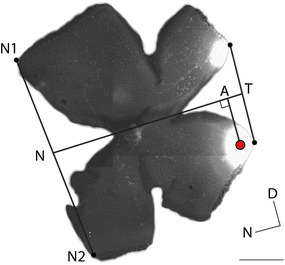
Illustration of projection method. The two end points of the nasal cut (*N1*,*N2*) are connected by a line, the centre of which is defined as the nasal pole (*N*). The same procedure is repeated on the temporal side, and the nasal (*N*) and temporal (*T*) poles are connected by a *line* (NT). The centre of the injection site (marked in *red*) is projected onto the nasotemporal axis, and the fraction of the distance is calculated as $|\mathrm {NA}|/|\mathrm {NT}|$, where $|\mathrm {NA}|$ is distance between N and A, and $|\mathrm {NT}|$ is distance between N and T. *Scalebar* 1 mm. Nasal (N) and Dorsal (D) directions are marked

To determine the location of the dye injection in the retina, it is dissected out and flattened so that it can be imaged (Fig. 1C) [10]. This is a delicate procedure and the flattening causes distortions and tears in the retina. The retinal location of the injection is then estimated from the image using the projection method [5, 6] or Retistruct [11]. In the projection method the location of the nasotemporal axis is first estimated based on the vestigial nictitating membrane at the nasal pole, and then the location of the injection site is projected onto the nasotemporal (NT) axis. The injection location is reported as a fraction of the NT axis, measured from the nasal pole (Fig. 2). The dorsoventral axis is calculated by orthogonal projection of the NT axis. This projection method uses a Cartesian coordinate system to describe a curved surface, which leads to distortions. To address this issue, the Retistruct program [11] was developed recently independently from earlier work using relaxation techniques [12]. Retistruct refolds the retina back onto a sphere to try and recover the original geometry. Internally it minimizes an energy function such that the stretching of the surface is as small as possible. Using Retistruct’s mapping of the flat image onto the sphere the injection site can be specified in the native three dimensional coordinates of the eye without distortions due to incompatible coordinate systems.

Retistruct is a significant improvement over the projection method, however, it still requires the retina to be dissected out and flattened. It also requires manual labelling of the outline of the retina. Here we propose a method that bypasses the need to dissect out the retina from the eye. Instead of using one image of the flattened retina, the IntactEye method uses two orthogonal images of the intact retina. With the help of two user-placed wire-frame spheres that are aligned with the pictures of the eye, IntactEye can calculate the location of the marked injection site in the 3D coordinate frame of the eye. The accuracy of the IntactEye method compares well with both the projection method and Retistruct. By avoiding the retinal ex vivo flattening, the acquisition is faster and we also reduce measurement artefacts and increase reproducibility and reliability. The IntactEye method is developed with the retina in mind, but we solve a general problem of locating a point in a three dimensional sphere from two images, which could have applications outside neuroscience.

## Implementation

In the first section we describe how to use the IntactEye method. We then describe the animal procedures used, and describe two other approaches of analysis that we compare to the IntactEye method. We also describe the new wedge coordinate system used to define the location of the retinal injection. The last section describes the verification of the IntactEye method using both known experimental retinal landmarks and synthetic data.

### Installation

IntactEye is free to download from [13]. An archived version of our program is also available from http://dx.doi.org/10.6084/m9.figshare.1605574. The zip file contains the source code and some example images. This article acts as the main documentation for the program. Unpack the zip file, then start MATLAB. In the MATLAB GUI click the “Set path” icon, and add the IntactEye directory to the path. Alternatively this can be done from the command line by executing addpath(’/your/path/to/IntactEye’); savepath.

### Preparation of images

To localise a retinal injection using IntactEye two images of the intact retina, with the injection site visible, must be prepared. We suggest one taken from the top looking down at the iris, and a second one taken from the side. For mouse we recommend a small cut at the vestigial nictitating membrane as a nasal marker. Make sure this cut is visible in at least one of the two images. The two images can be loaded separately or as a part of a composite image. The software reads images in tiff, png and jpeg format. The images need to have the same magnification and either have the same height or width so they can be automatically merged.

### How to use the software

A video of the following four-step procedure can be found on YouTube [14].

*Step 1* Open up Matlab and type IntactEye to start the program. In the user interface click “Load Image”. This brings up a dialog box to select which composite file to load. Alternatively, “Load Images” allows you to load the two views from two separate files. The images of the eye are displayed with two wire-frame spheres overlaid. The “Left Eye”/“Right Eye” button lets the user select if it is a left or right eye that is being analysed; this switches the dorsoventral (DV) axis accordingly.

**Fig. 3:**
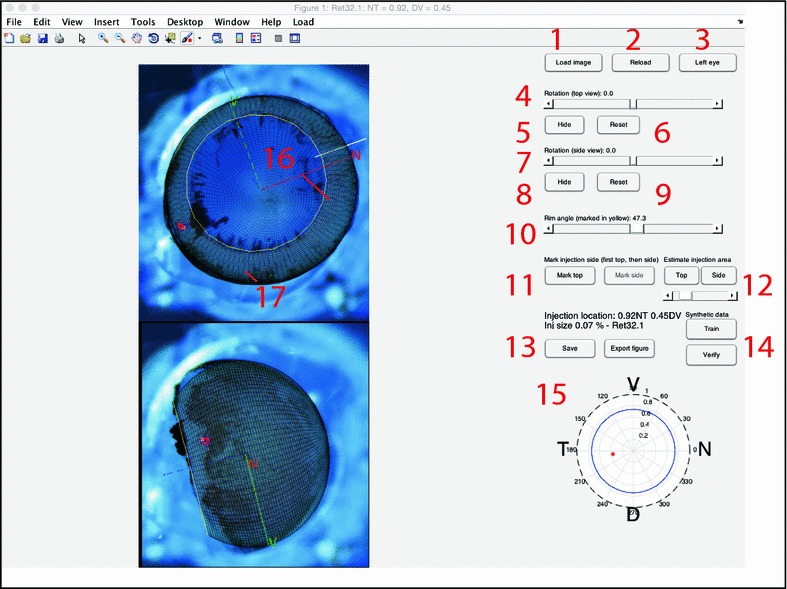
Screenshot of IntactEye being used used to mark up the eye and injection. The two images have a wire-frame overlaid such that the axes can be reconstructed. *1* Load image. *2* Reload a previously saved file. *3* Toggle *left* and *right eye* coordinate system. *4* Slider to rotate the eye around the centre axis in *top view*. *5* Hide wire-frame for top view. *6* Reset *top view*. *7*–*9* Same as *4*–*6*, but for side view. *10* Slider to adjust rim angle (*yellow circle in figure*). *11* Mark injection in *top* and *side views*. *12* Estimate area of injection side from *top* or *side view*. Slider adjusts threshold. *13* Save results to MAT file and export figures. *14* Controls to generate synthetic data and verify user accuracy on the synthetic data. *15* Flat view of injection site location, showing *spherical coordinates*. The *red dot* is *centred* on the location and drawn to scale to reflect the approximate area of the injection. *16* Retinal rim. *17* Injection location. Coordinate axes of the nasal and ventral axis are shown. As a visual guide the axes lines are shown as *solid* if they are above the image plane, and *dashed* if below it. The optic disc is visible close to the centre of the eye in the *top view*. A *thin white line* just under has been drawn by the experimentalist in the image to mark the nasal injection site, barely visible under the nasal axis in the *top view*

*Step 2* Using the computer mouse, the wire-frame spheres can be interactively resized and repositioned (Fig. 3). The left mouse button is used when the sphere is resized (by clicking and dragging on the axis handles) or moved (by clicking and dragging the centre). The right mouse button is used when rotating the wire-frame sphere. There are also two sliders to adjust the rim angle and rotate the eye around its central axis. If a nasal cut was made, it must be aligned with the nasal marker. The wire-frame spheres can be temporarily hidden by clicking the “Hide” button. Clicking the button again shows the wire-frame spheres. If the nasal cut is only visible in the top view, then the injection can sometimes be used to help align the second sphere.

*Step 3* Once the spheres match the images of the eye, the next step is to mark the injection site. Start by marking it in the top view by clicking “Mark top” and then clicking on the injection location. A line is then displayed in the side view with the possible locations. Click “Mark side” and place a mark in the side view and the program computes the nasal and dorsal positions.

*Step 4* Click “Save” to create a MAT file with the eye image as well as the location of the wire-frame and injection. This information can be accessed by using the “Reload” button to return to a previously saved state. The “Export Figure” button saves a 2D picture of the injection location (and, optionally, extent) in polar coordinates, as well as an image with the wire-frame spheres overlaid on the original images.

### Experimental protocol

To test the IntactEye method we collected two sets of retinal images from mouse. First we dissected out and imaged the intact retina, then we performed additional dissection to acquire images of the flattened retina that could be analysed for comparison.

C57/Bl6J mice were housed at the Chronobiotron, CNRS UPS 3415, Strasbourg, under a 12/12 h light/dark cycle. All procedures were in accordance with European community (2010/63/EU) guidelines. Official agreement number for animal experimentation was 01831.01 (MR). Standard laboratory rodent food and water were available *ad libitum*.

Neuronal lipophilic tracer injection and dissection procedure were performed as previously described [15]. Small volumes (30–50 nl) of 1,1-dioctadecyl-3,3,3,3-tetramethylindocarbocyanine perchlorate (DiI) were injected in the left retina of P7-P8 C57/Bl6 mice. DiI was dissolved in dimethylformamide, loaded into a pulled glass pipette, and pressure injected into the retina using a Picospritzer. After 16 to 18 h, animals were euthanized by a lethal dose of pentobarbital (800 $\upmu$g/g), perfused with 4 % PFA and decapitated. The head skin was removed and a cut was made at the level of the nictitating membrane (flap) indicating the nasal pole of the retina. Oculomotor muscles were then cut and fine tilted forceps were used to enucleate the eye. Cornea, sclera and pigmented epithelium were then delicately removed using tweezers, revealing the retina. The intact retina was then photographed from a top-down and a side view, showing the injection site (Fig. 1A, B) using a Zeiss binocular coupled to digital camera. These can then be analysed using IntactEye.

The projection method (Fig. 2) and Retistruct both require additional steps to be performed. For flat-mount dissection (Fig. 1C), cardinal cuts (temporal, dorsal and ventral) were performed according to the original nasal cut. The retina was then flattened on a coverslip and mounted onto a glass slide. A picture at low magnification of the full flat-mounted retina was taken using Zeiss Axioskop 2. These flattened images can then be used with the projection method or Retistruct.

### Projection method

The projection method [5, 6] is a standard method used to estimate the location of the nasotemporal axis based on the nasal cut and the optic disc (Fig. 2). In the image of the flattened retina, the two nasal points (N1, N2) that were separated by the cut are reconnected by a straight line. The middle of this line is defined as the nasal pole (N). The same procedure is applied to find the temporal pole (T). A second line is drawn from N to T. This line (NT), which runs from the nasal pole to the temporal pole via the optic disc, defines the nasotemporal axis. The injection location is projected onto this line and the position (A) reported as a fraction of the whole nasotemporal axis ($|\mathrm {NA}|/|\mathrm {NT}|$, where $|\mathrm {NA}|$ is the length of the line NA).

### Retistruct method

The Retistruct method [11] is used here as a control to evaluate our method. Retistruct’s starting point is a flattened retina. The user provides the algorithm with a mark-up of the periphery of the retina indicating where the cuts and tears were. This information is used to fold the flattened retina back onto a sphere. The refolding takes into account the rim angle and is done so as to minimize the amount of stretching and compression of the retinal image. The location of the injection is then calculated in the native 3D space of the eye.

### IntactEye method

**Fig. 4:**
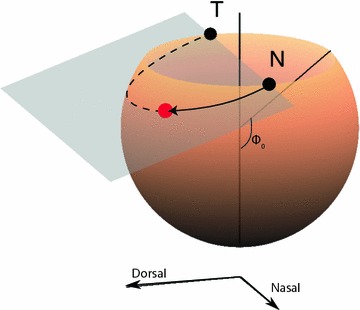
The wedge coordinate system. The coordinate of each point can be found by taking a plane through the nasal pole (N), temporal pole (T) and the point of interest (*red dot*). The intersection of the plane and the sphere forms an arc (*solid arrow* and *dotted line*). The nasotemporal coordinate is the fraction (*f*) of the distance along the arc from the nasal to the temporal pole. The rim angle ($\phi _0$) is used when calculating the wedge coordinates. The dorsoventral coordinate is defined analogously

By using two images taken from different angles, the location of an injection mark in an eye can be found without flattening the retina. To estimate the view angle for each of the images, the user needs to indicate where the eye is positioned and which way it is turned. This is done by placing a wire-frame eye-ball over the image of the intact retina. The controls in the program allow the user to rotate the eye along the three axes and to adjust the lengths $r_x$, $r_y$ and $r_z$ of the semi-axes of the ellipsoid. The rim angle (Fig. 4) of the eye-opening is manually adjusted to improve the fit between the wire-frame model and the image of the eye. By marking the position of the injection in the top image ($x_\mathrm {top},y_\mathrm {top}$), the location of the injection site can be narrowed down to a line segment between $p_1 = (x_\mathrm {top},y_\mathrm {top},r_\mathrm {max})$ and $p_2 = (x_\mathrm {top},y_\mathrm {top},-r_\mathrm {max})$ perpendicular to the imaging plane, where $r_\mathrm {max}$ is the largest radius of the ellipsoid. The view transform is

1 $$\begin{aligned} T(a) = T_\mathrm {rot} a +\mathbf {t}_\mathrm {trans}, \end{aligned}$$

where for rotation $\vartheta _x,\vartheta _y,\vartheta _z$ around the *x*, *y*, *z*-axis the rotation matrix is

2 $$\begin{aligned} T_\mathrm {rot} = \begin{pmatrix} 1 &{} 0 &{} 0 \\ 0 &{} \cos \vartheta _x &{} -\sin \vartheta _x \\ 0 &{} \sin \vartheta _x &{} \cos \vartheta _x \end{pmatrix} \begin{pmatrix} \cos \vartheta _y &{} 0 &{} \sin \vartheta _y \\ 0 &{} 1 &{} 0 \\ -\sin \vartheta _y &{} 0 &{} \cos \vartheta _y \end{pmatrix} \begin{pmatrix} \cos \vartheta _z &{} -\sin \vartheta _z &{} 0 \\ \sin \vartheta _z &{} \cos \vartheta _z &{} 0 \\ 0 &{} 0 &{} 1 \end{pmatrix} \end{aligned}$$

and $t_\mathrm {trans}$ is a vector. The inverse transform is

3 $$\begin{aligned} T^{-1}(b) = T_\mathrm {rot}^{-1}(b - \mathbf {t}_\mathrm {trans}) \end{aligned}$$

and the line representing possible locations for the injection in the eye frame runs from $q_1 = T^{-1}(p_1)$ to $q_2 = T^{-1}(p_2)$. By plotting this line in the second view overlaid on the eye, we can quickly establish which point in 3D space corresponds to the injection. This method relies on accurately placing the two wire-frame eyes on top of the real images.

Internally the program stores the coordinates of the injection ($x_\text {inj},y_\text {inj},z_\text {inj}$) in Cartesian coordinates in the coordinate frame of the eye (*E*). The program also stores the viewing transforms for each of the two views, which tracks the 3D rotation and the translation of the spheres.

### Wedge coordinate system

A coordinate system which defines arcs running from the nasal to the temporal poles, along which the fractional distance *f* from nasal to temporal can be measured. A fractional distance from the dorsal to the ventral poles can be defined analogously. This section is presented for information only; the user can use the program without studying the new coordinate system.

Assume the retina is oriented as shown in Fig. 4, with the rim lying at a colatitude of $\phi _0$ measured from the south pole. Each point on the surface of the curtailed sphere can be reached by a system of coordinates $(\psi , f)$ where $\psi$ is the angle to the vertical made by a plane passing through the nasal and temporal poles and *f* is the fractional distance along the circle defined by the intersection of this plane and the curtailed sphere. Assuming a sphere of unit radius, the forward transformation from wedge coordinates $(\psi , f)$ to Cartesian coordinates (*x*, *y*, *z*) is:

4 $$\begin{aligned} &x = \rho \sin (\alpha _0 + f(2\pi - 2\alpha _0))\nonumber \\ &y = y_0 - \rho \sin \psi \cos (\alpha _0 + f(2\pi - 2\alpha _0)) \nonumber \\ &z = z_0 + \rho \cos \psi \cos (\alpha _0 + f(2\pi - 2\alpha _0)) \nonumber \\ \end{aligned}$$

where

5 $$\begin{aligned}& \rho = \sqrt{\sin ^2\phi _0 +\cos ^2\phi _0\cos ^2\psi } \nonumber \\& y_0 = -\sin \psi \cos \psi \cos \phi _0 \nonumber \\ &z_0 = -\sin ^2\psi \cos \phi _0\nonumber \\ &\alpha _0 = -\arcsin \left( \frac{\cos \phi _0}{\rho }\right) \end{aligned}$$

Here $\rho$ is the radius of the circular arc whose centre is $(0, y_0, z_0)$, and $\alpha _0$ is the value of the angular parameter along the circle at the rim.

To invert (*x*, *y*, *z*) back to $(\psi ,f)$ the following equations are used:

6 $$\begin{aligned}& \psi = \text {atan}(y, -\rho \cos \phi _0 - z) \nonumber \\ &v = -(y - y_0)\sin \psi + (z - z_0)\cos \psi \nonumber \\ &\alpha = \text {atan}(x, v) \nonumber \\& f = (\alpha - \alpha _0)/(2\pi - 2\alpha _0) \end{aligned}$$

where

7 $$\begin{aligned} \text {atan}(y,x) = {\left\{ \begin{array}{ll}\arctan (y/x) - \pi &{}\quad \text {if } x > 0, y < 0 \\ \arctan (y/x) + \pi &{} \quad\text {if } x>0, y> 0\\ \arctan (y/x) &{} \quad\text {otherwise} \\ \end{array}\right. } \end{aligned}$$

Internally, IntactEye uses Cartesian coordinates, and the inverse transformation (Eqs. 6, 7) is used to generate the output. The forward transformation (Eqs. 4, 5) is given as reference.

### Injection area estimation

As well as reporting the location of the centre of the retinal injection, the area can also be estimated from either the top view or the side view, as long as the entire injection site is visible. IntactEye automatically converts the image from RGB into the L*A*B* colour space, which has one value for lightness and the other two for colour hues. The lightness value is discarded. If the injection centre in the image is located at (*p*,*q*) then the distance in colour space for a pixel at (*i*, *j*) is calculated as

8 $$\begin{aligned} d_{ijpq} = \sqrt{(A_{ij} - A_{pq})^2 + (B_{ij}-B_{pq})^2} \end{aligned}$$

where $A_{ij}$, $B_{ij}$, $A_{pq}$, $B_{pq}$ are the hue values. The image is thresholded (default threshold 5) so that only the parts with similar colour hue to the injection centre are selected. To find the corresponding points on the sphere, IntactEye takes a line through each injection pixel, perpendicular to the image plane, and finds its intersection with the eye ellipsoid. This is done by calculating

9 $$\begin{aligned} v = \text {abs}\left( x^2/r_x^2 + y^2/r_y^2 + z^2/r_z^2-1\right) \end{aligned}$$

(where $r_x,r_y,r_z$ are the lengths of the semiaxes of the ellipsoid) for 2000 points on the line, and picking the one with the smallest *v*. The fraction of the total area of the eye is reported as the injection size. The injection area is calculated using MATLAB’s built in alpha hull [16] function (alphaShape); this functionality requires matlab version 2014b or newer; older versions of matlab will not calculate the area of injections, but can still calculate the location.

### Synthetic data

We generated a set of synthetic data to train the user, and also to verify the accuracy of the method. The length of the semi-axes of the ellipsoid representing each eye are drawn from a normal distribution ($1400\pm 70\,\upmu \text {m}$), corresponding to a P12 mouse eye [11]. The rim angle defining the opening was sampled from a normal distribution ($53^\circ \pm 3^\circ$). For the top and side views, the view angle and location was varied approximately within a range of $\pm \,20^\circ$ around the x and y axis (normal distribution ($0^\circ \pm 9^\circ$) and freely rotating around the z-axis. The nasal cut was marked with a M. The location of the injection centre (spherical coordinates $\theta _\mathrm {inj} \in [0^\circ ,360^\circ ],\phi _\mathrm {inj} \in [0^\circ ,180^\circ ]$) was randomized from a uniform distribution. The injection site was represented by 100 points, with spherical coordinates sampled from a normal distribution centered on the injection centre, and with standard deviation $6^\circ$. Any points placed above the rim were discarded. Three observers were asked to estimate the location of the centre of the injection site in synthetic data. The user-provided location and the known location were then compared.

### Locating the optic disc

By using the location of the optic disc as a known landmark close to the geometric centre of the eye [17] we can estimate the accuracy of the Retistruct and IntactEye. Images where the optic disc was not clearly visible were excluded from this verification step.

## Results and discussion

We have created a software package named “IntactEye” to calculate the location of a retinal injection from two orthogonal pictures of an intact retina. IntactEye lets the user manually place two reference wire-frame spheres on the images of the eye. The program reports the nasotemporal and dorsoventral coordinates of the injection as a fraction along the respective axes using our wedge coordinate system (Fig. 4). The injection size is reported as a fraction of the area of the retina.

**Fig. 5:**
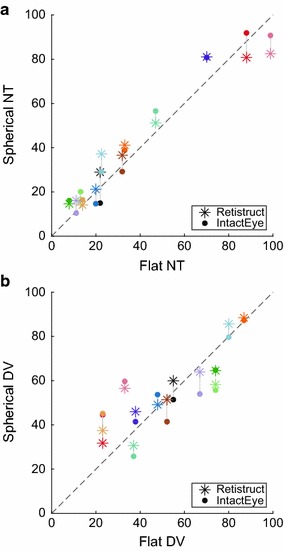
Comparison of nasotemporal and dorsoventral coordinates for retinal injections derived from three different methods. **a** The coordinates on the flattened retina from the projection method are shown on the x-axis and the NT coordinates on the spherical eye from both Retistruct and the IntactEye method are shown on the y-axis. **b** As in **a**, but for the DV axis. Points estimated from the same eye are shown in the same colour and connected by a *thin grey line*. The *diagonal line* shows the case when the coordinates in the flat and spherical coordinate system agree

**Table 1:** Pearson correlation coefficient for NT (DV) coordinates between the three methods for data shown in Fig. 5

	Retistruct	IntactEye
Projection	0.97 (0.88)	0.98 (0.74)
IntactEye	0.98 (0.92)	–

To compare IntactEye with the projection method and Retistruct, a set of wild type mouse retinas (N=11) which had been imaged both before and after flattening was analysed. Fig. 5 compares the position estimates from the three methods, for the nasotemporal axis (A) and the dorsoventral axis (B). We see that there is a good correspondence between all three measures, but there is a larger variation in the dorsoventral coordinates than in the nasotemporal coordinates (compare Pearson correlation coefficients in Table 1). For the projection method the nasal cut and flattening will cause a larger distortion of the dorsoventral axis than the nasotemporal axis due to the direction of the cut.

**Fig. 6:**
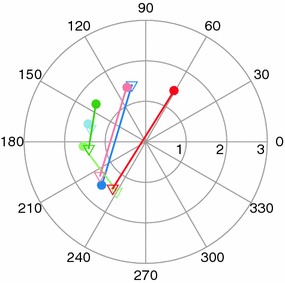
Estimating the precision of the IntactEye method by locating the optic disc. The optic disc is located in the centre of the eye and was visible in 6 out of 11 retinas. For each of the six retinas, the optic disc location was estimated in polar coordinates using IntactEye (*triangles*) and Retistruct (*filled circles*). Lines connect two estimates from the same retina. In all six cases, The optic discs were located within 2$^\circ$ of the geometric centre; by comparison the rim is located at 127$^\circ$ and so the error in locating the optic disc was under 2 %

We assess the accuracy of the IntactEye using two methods. First we used the location of the optic disc, a known retinal landmark. The optic disc was visible in six out of eleven images investigated. By marking the optic disc in the images using IntactEye we estimated the location as $49\pm 1\,\%$ (mean $\pm$ SD) nasotemporal and $49\pm 2\,\%$ dorsoventral (Fig. 6). This is comparable to the results from Retistruct when applied to the same retinas: $48\pm 1\,\%$ nasotemporal and $51\pm 2\,\%$ dorsoventral (Fig. 6).

**Fig. 7:**
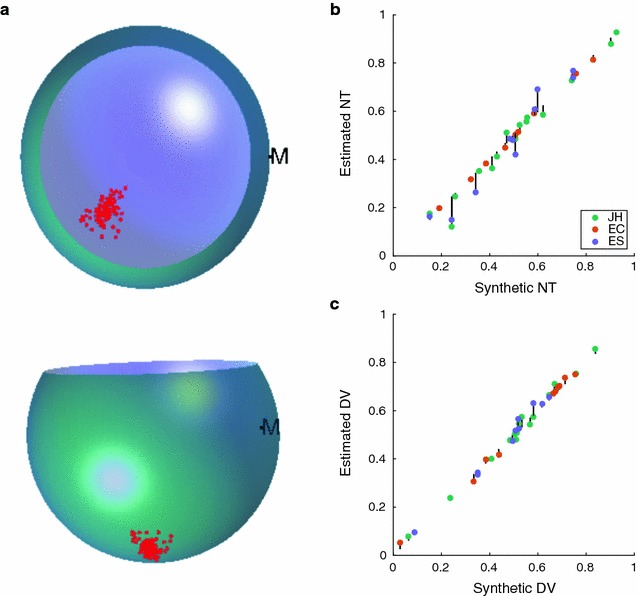
Verification of IntactEye using synthetic data. **a** Example of synthetic data used in the test. The injection site is represented by 100 randomized points sampled from a normal distribution centred around the injection site. The nasal cut is marked with the *letter M*. **b**, **c** Known NT/DV coordinates of synthetic injections plotted against user-estimated NT/DV coordinates from IntactEye. *Vertical black lines* indicate deviation from correct location

We also created synthetic images of eyes with label injections where the exact location was known. This allowed us to assess the variability with the IntactEye method that comes from fitting the spheres to the image of an idealised eye. Three observers each marked a unique set of synthetic data. Figure 7 shows the known NT (DV) coordinates on the x-axis and the corresponding estimates on the y-axis. We see a good correspondence between the true location and the estimated position in the synthetic data (Pearson correlation coefficient 0.98 and 0.99 for NT and DV axis). These images are relatively clean, lacking deformations of the eye and imperfections such as debris and limited depth of field. However, it provides us with images where the centre of the injection is known and allows us to test misalignment of the wire-frame spheres onto the images of the eye.

### Limitations

Each point that is localised using IntactEye must be uniquely identified in both images of the retina. Because of this limitation the IntactEye method is best suited for finding single injections and other distinct landmarks. If more than one distinct region is labelled with the same dye, it may be difficult to uniquely identify the same region in the two different images. This is known as the correspondence problem in stereo vision [18]. This may limit the use to anterograde injections where the retinal marking is focused. For retrograde injections in animals where the label is spread over a large region of the retina (e.g. when topographic maps are perturbed, such as Figure 4 of [19]), it is currently better to dissect the retina and then use a program like Retistruct, which analyses the entire retina and can generate density estimates.

### Future work

By using the IntactEye method we can locate the centre of a single injection from images taken from any two distinct views where the injection is visible. The program currently only tracks one injection, but could be extended to handle multiple injections of different coloured dyes. This, together with the area estimation, might allow the processing of focal retrograde injections as well, as long as they are wholly visible in the images, potentially by allowing for additional views of the retina.

The manual placement and alignment of the wire-frames takes a few minutes, and is a candidate for automisation. However currently the time-intensive part is the data acquisition; for example dissecting out the retina and flattening procedures takes 10–15 min.

An alternative method to our current problem of locating an injection site might be to image the entire eye to generate e.g. a z-stack of images. Although this would avoid the need for any retinal dissection, it does not in itself solve the subsequent problem of registering the retinal location in a standard coordinate space. Therefore, generating volumetric images of eyes would not allow the injection sites to be compared meaningfully with each other. By contrast, if a z-stack is already available for an eye, it should be possible (resolution permitting) to extract two orthogonal images suitable for our program.

## Conclusions

We have developed a method that uses two images of an intact retina to derive the location of a retinal injection. This bypasses the need to cut and flatten the retina, improving the accuracy of the localisation as the tissue undergoes less distortion, and saving the experimentalist time. By analysing the data in a coordinate system native to the shape of the eye, we avoid the problem of representing a spherical structure in a flat coordinates. To verify our results we analysed the data in three different ways. We found good correspondence between the coordinates of our IntactEye method and of Retistruct, which uses flattened images and then folds them back onto a sphere. Both methods produce coordinates in the native curved space of the eye. We have solved the abstract problem of deducing the location of a point on a spherical object from two images, and implemented it for our use analysing mouse retinae, where there are no discernable landmarks for orientation within the retina. It can however be used in other species where retinal landmarks are available, such as the corneal marks in goldfish. Furthermore, this technique might have clincial applications to human retinas, where a reliable coordinate system is required for describing retinal locations, based for example on MRI images. Finally, as our approach is to treat the retina as a simple geometrical, rather than neuronal object, we imagine this technique can be applied straightforwardly to a wide range of fields outside of neuroscience.

## Availability and requirements

Project name: IntactEye. Project home page: http://github.com/hjorthmedh/IntactEye. Archived version available at: http://dx.doi.org/10.6084/m9.figshare.1605574. Operating system(s): Any platform that runs MATLAB 2014b or later (tested on mac, linux and windows). Programming language: MATLAB. Other requirements: None. Any restrictions to use by non-academics: GPL/MIT.

## Abbreviations

N: nasal
T: temporal
D: dorsal
V: ventral
NT: nasotemporal axis
DV: dorsoventral axis
